# Bioconversion of Raw Glycerol From Waste Cooking-Oil-Based Biodiesel Production to 1,3-Propanediol and Lactate by a Microbial Consortium

**DOI:** 10.3389/fbioe.2019.00014

**Published:** 2019-02-18

**Authors:** Xiao-Li Wang, Jin-Jie Zhou, Ya-Qin Sun, Zhi-Long Xiu

**Affiliations:** School of Life Science and Biotechnology, Dalian University of Technology, Dalian, China

**Keywords:** low-quality raw glycerol, microbial consortium, 1, 3-propanediol, lactate, non-sterile conditions

## Abstract

Waste cooking oil (WCO) is a sustainable alternative to raw vegetable oils and fats for biodiesel production considering both environmental and economic benefits. Raw glycerol from WCO-based biodiesel production (GWCO) is difficult to utilize via biological method, as multiple toxic impurities have inhibitory effects on microbial growth especially for pure cultures. In this work, four microbial consortia were selected from activated sludge by 30 serial transfers under different conditions. The obtained consortia exhibited lower diversity and species difference with the transfers. The consortium LS30 exhibited unique advantages for bioconversion of GWCO to 1,3-propanediol (1,3-PDO) and lactate (LA). Moreover, the fermentation could be performed economically under microaerobic and non-sterile conditions. The consortium consisted of 57.97% *Enterobacter* and 39.25% *Escherichia* could effectively convert 60 g/L GWCO to 1,3-PDO and LA in batch fermentation. In addition, this consortium exhibited better tolerance to fatty acid-derived crude glycerol (100 g/L), which demonstrated that specific toxic impurities in GWCO did pose a great challenge to microbial growth and metabolism. In fed batch fermentation, 27.77 g/L 1,3-PDO and 14.68 g/L LA were achieved. Compared with the consortium, a long lag phase in cell growth associated with a decreased glycerol consumption was observed in four single-strain fermentations. Furthermore, neither the consortium DL38 with excellent glycerol tolerance nor consortium C2-2M with high yield of 1,3-PDO could effectively transform GWCO into valuable products. The results demonstrated that the selected microbial consortium has the advanced adaptability to the toxic impurities in GWCO compared with other reported consortia and isolated single strain. This process can contribute to added-value use of GWCO.

## Introduction

Biodiesel, as a biodegradable and non-toxic substitute for petroleum diesel, can be obtained by the transesterification of WCO instead of conventional raw vegetable oils or animal fats (Singh and Singh, 2010; Su and Guo, 2014; Guabiroba et al., 2016). The amount of WCO, that was disposed in water and land as waste biomass was as much as 15 million tons annually in the world. On the other hand, WCO has profound significance for the development of biodiesel production (Lam et al., 2010; Mansir et al., 2018). According to Brazilian biodiesel producers association APROBIO, 980 mL biodiesel can be produced from 1L WCO (Rodrigues et al., 2018). WCO has many merits for the biodiesel production with respect to economical and social viability (Kulkarni and Dalai, 2006; Yaakob et al., 2013). Firstly, compared to the traditional raw edible feedstocks (including oil from rapeseed, soybean, palm, sunflower, and coconut), the utilization of WCO is able to reduce the cost of biodiesel production by 60–70% (Rodrigues et al., 2016). Secondly, as a kind of waste biomass, WCO can be reused to produce environment-friendly products, relieving the environmental pressure caused by its random discharge and the phenomenon of returning to the kitchen table. Thirdly, replacing raw edible oil with WCO can avoid the competition of land between energy and food (Lee et al., 2014; Guabiroba et al., 2016). Therefore, many process explorations and feasibility analyses of biodiesel production from WCO have been conducted and reported (Zhang et al., 2003a,b; Sirisomboonchai et al., 2015).

Raw glycerol is an accumulated by-product of biodiesel production. Crude glycerol accounts for 10% by weight of biodiesel and is already large surplus as a result of explosive growth of biodiesel production (Zhou et al., 2018). Raw glycerol from WCO-based biodiesel (GWCO) contains a variety of impurities, including methanol, salts, soaps, residual fatty acids, heavy metals, aflatoxin as well as benzopyrene (Moon et al., 2010; Rodrigues et al., 2018). The purification process of crude glycerol is unprofitable because of the low price of refined glycerol. Hence, the direct utilization of raw glycerol is vitally important in view of raw glycerol valorization, as well as sustainable development of global biodiesel production industry (Chatzifragkou and Papanikolaou, 2012; Monteiro et al., 2018). Interestingly, glycerol is a versatile starting molecule in bio-chemicals production, such as 1,3-propanediol (1,3-PDO), 1,2-propanediol, 2,3-butandiol, dihydroxyacetone, 3-hydroxypropionic acid, succinic acid, lactate (LA), ethanol, and polyhydroxybutyrate (Sun et al., 2018). Both oxidation and reduction pathways are indispensable in microbial glycerol assimilation so as to balance energy couple (ADP/ATP).

1,3-PDO, a potential C3 dihydroxy compound, is widely used for the generation of textiles, coating materials, plastics, medicine, and other industries (Lama et al., 2017). The most popular application of 1,3-PDO is as the monomer of polytrimethylene terephthalate (PTT). PTT embodies many excellent properties of all fibers, such as softness, fluffiness, stain resistance, and normal temperature dyeing. Therefore PTT is regarded as an advanced material an alternative to the conventional fibers (Rossi et al., 2012; Zhang et al., 2017). The production of 1,3-PDO from raw glycerol by microbial fermentation has aroused worldwide attentions concerning the environmental and sustainable development of the society (Liu et al., 2010; Monteiro et al., 2018; Zhou et al., 2018). Another important compound, LA can also be produced through glycerol fermentation and has been meaningfully applied to food and food related industries. Particularly, LA can form polylactic acid polymer (PLA) which is one of the most sustainable polymers for biodegradable and biocompatible plastics (Chen et al., 2014; Hu et al., 2016; Mitrea et al., 2017). The production of 1,3-PDO is from reducing branch in glycerol metabolism. In order to increase the efficiency of raw glycerol conversion appropriately, LA, as a meritorious product from oxidative branch, is acceptable (Xin et al., 2017). Natural microorganism species, which possess the ability to produce 1,3-PDO from glycerol, involve *Klebsiella, Clostridium, Enterobacter, Citrobacter*, and *Lactobacillus* (Saxena et al., 2009; Celinska et al., 2014; Zhou et al., 2017). Among them, *Klebsiella pneumoniae* (Yang et al., 2016; Xin et al., 2017) and *Clostridium butyricum* (Dams et al., 2016; Zhou et al., 2017) get more attention considering their high productivity and concentration of 1,3-PDO. However, pure strains show fairly poor tolerance to raw glycerol degradation. Especially glycerol from biodiesel contains much impurities (Mu et al., 2006; Moon et al., 2010; Chatzifragkou and Papanikolaou, 2012; Yang et al., 2016). Therefore, GWCO, which contains more unspecified impurities, would be more difficult to be utilized than other crude glycerol by pure cultures.

Conversely, microbial consortium exhibits strong resistance to complex substrate, simplified operating conditions, adjustable metabolism and fewer by-products in glycerol fermentation (Dietz and Zeng, 2014; Sabra and Zeng, 2014; Jiang et al., 2017a,b; Zhou et al., 2017, 2018). The microbial consortium DL38 dominated by *Klebsiella* could endure an initial glycerol concentration of 200 g/L more than predicted value (188 g/L) (Zeng et al., 1994; Jiang et al., 2017a). The microbial consortium C2-2M, mainly consisted of *Clostridiaceae*, can produce the highest 1,3-PDO concentration of 82.66 g/L in fed-batch fermentation. This consortium maintains strong and stable 1,3-PDO productivity in batch, fed-batch and continuous fermentation (Zhou et al., 2017, 2018). It is worth to mention that the microbial consortium selected by Dietz et al. (Dietz and Zeng, 2014) is able to grow and produce 1,3-PDO up to 70 g/L without nitrogen gassing and the addition of yeast extract in medium under non-sterile conditions. However, in all these studies, raw glycerol is derived entirely from raw edible-oil-based biodiesel or hydrolysis of vegetable oils and fats. So far, as for the satisfied utilization of GWCO, only few reports demonstrated its practicability in hydrogen production combined with the highest 1.3-PDO concentration of 5.85 g/L, which is far inferior to the utilization of crude glycerol from other sources (Pachapur et al., 2016; Rodrigues et al., 2018). Therefore, high efficiency production of 1,3-PDO with GWCO is of great significance. Moreover, most fermentation processes have no advantages because of high consumption of precious fresh water and energy caused by energy-intensive sterilization processes (Yue et al., 2014). Industrial biotechnology should play a role in the sustainable development of human society. It is worth noting that 97% of the water on earth is seawater. With this in mind, a microbial consortium, which could consume GWCO in sea water, has great value for industrial application. In that case, a fermentation process would be exploited for simple operations and lower production cost.

In the present study, four microbial consortia that represent structural diversity were obtained by acclimating them to raw glycerol from different sources. Then, in batch and fed-batch fermentations, conversion of raw glycerol from WCO-based biodiesel production to valuable products (1,3-PDO and LA) under microaerobic and non-sterile conditions by the selected microbial consortium was investigated. Moreover, the adaptability of the selected microbial consortium, single strains isolated from the consortium and other representative microbial consortia to GWCO were evaluated.

## Materials and Methods

### Raw Glycerol Source

Two types of glycerol were used in this work. One is the byproduct of biodiesel production from WCO with sulfuric acid (H_2_SO_4_) as catalyst. The characterization of this raw glycerol is presented in Table 1. Furthermore, some trace metal ions were determined by inductively coupled plasma mass spectrometry (ICP-MS) as show in Supplementary Table 1. The other crude glycerol is from hydrolysis of raw vegetable oils (named by GHVO). The quality and origin of the GHVO was described by Zhou et al. (2017). The glycerol concentration presented in this study stood for the absolute glycerol content of medium without regard to impurities.

**Table 1:** The characterization of the raw glycerol from biodiesel production using waste cooking oil.

**Parameters**	**Methods**	**Value**
pH	pH meter	7.08
Glycerol (m/m) %	HPLC	49.30
Moisture and volatile matter (%)	GB/T 14489.1-2008	16.44
Soap (m _sodiumoleate_/m) (%)	GB/T 5533-2008	12.10
Salts (m _Na2SO4_/m) (%)	Electrical conductivity meter	9.72
Ash (m/m) (%)	GB/T 13216-2008	10.65
Aflatoxin B1 (ppb)	Immunochromatography strip test	2.5–5
Methanol (m/m) (%)	HPLC	1.2
Acetate (m/m) (%)	HPLC	3.9
1,3-PDO (m/m) (%)	HPLC	3.8

*Immunochromatography strip test from Shenzhen Finder Biotch Co.,Ltd*.

### Media

The seed media were used for selection of the consortia and seed culture. Seed medium components (Jiang et al., 2017a) contained: glycerol 20 g/L, K2HPO4·7H2O 4.454 g/L, KH2PO4 1.3 g/L, (NH4)2SO4 2.0 g/L, MgSO4·7H2O 0.2 g/L, yeast extract 1.0 g/L, 1 mL/L Fe2+ solution (HCl 4 mL/L, FeSO4·7H2O 5 g/L), 1 mL/L CaCl2 solution (20 g/L), 2.0 mL/L trace element A solution (HCl 0.9 mL/L, CuCl2·2H2O 20 mg/L, ZnCl2 70 mg/L, MnCl2·4H2O 100 mg/L, H3BO3 60 mg/L, CaCl2·6H2O 200 mg/L, NiCl2·6H2O 25 mg/L, NaMoO4·2H2O 35 mg/L), CaCO3 5.0 g/L. In seed media for microbial consortia culture, two types of raw glycerol were used as the carbon source; Water or tap water were used to prepare the media according to experimental objective. Four types of seed medium formulations were used for selection of microbial consortia. The seed media A and D were composed of GWCO as the main carbon source and prepared with tap water (A) or seawater (D). The seed media B and C were composed of GHVO as the main carbon source and prepared with tap water (B) or seawater (C). Glycerol concentration is 20 g/L in all seed media.

The fermentation media were performed on basis of Jiang et al. (2017a). Fermentation medium used for 1,3-PDO production contained: KH_2_PO_4_ 1.36 g/L, (NH_4_)_2_SO_4_ 6.61 g/L, MgCl_2_·6H_2_O 0.26 g/L, CaCl_2_·6H_2_O 0.29 g/L, citric acid 0.42 g/L, yeast extract 2.0 g/L, 5 mL/L trace element B solution (CuCl_2_·2H_2_O 0.47 g/L, ZnCl_2_ 0.68 g/L, MnCl_2_·4H_2_O 0.17 g/L, H_3_BO_3_ 60 mg/L, NaMoO_4_·2H_2_O 5 mg/L, FeCl_2_ · 4H_2_O 3.97 g/L, CoCl_2_·6H_2_O 0.47 g/L, HCl 10 mL/L). In fermentation media, the two raw glycerol types were used as substrates with different glycerol concentrations as experiment requirement.

In pure culture, the seed medium A was used. The solid medium used for single strain separation was the same as the seed medium with the addition of 12 g/L of agar and no CaCO_3_ added.

### Microbial Consortia Culture

Seven consortia were involved in this paper, five of which were obtained through screening. And the other two named C2-2M (Zhou et al., 2017) and DL38 (Jiang et al., 2017a) were preserved in our lab. The inoculated activated sludge sample used in this work came from Lingshui River Sewage Treatment Co., Ltd., Dalian, China.

The seed cultures were carried out in 250-mL sealed serum bottles with the seed media volume of 100 mL. Seed media were always sterilized and nitrogen was bubbled into the serum bottle to create an anaerobic environment before inoculation. Then the seed cultures were incubated at 37°C with a fixed shaking rate (200 rpm). Inoculation and sampling were operated by sterile syringes after finishing seed cultures (12–80 h).

The microaerobic batch and fed-batch fermentations were performed in a 5 L bioreactor (Baoxing Biotech, Shanghai, China) with the working volume of 2 L. Before batch and fed-batch fermentations, the stock cells were transferred into seed media and cultivated for 14 h to activate the culture. Then the inoculum culture was accomplished by seed media with 2% (v/v) of the activated broths for another 12 h. Afterwards, the seed broths were inoculated into a prepared bioreactor with 10% (v/v). The pH was controlled at 7.0 by automatic addition of 5 M NaOH. The fermentation was performed at a constant temperature of 37°C with an agitation rate of 200 rpm. In the whole microaerobic fermentations process, no nitrogen or air was pumped into the bioreactor. Therefore, the medium was in a normal dissolved oxygen (DO) state at the beginning of fermentation. During fermentation, the DO concentration will gradually decrease or even become zero. In anaerobic fermentations, the anaerobic environment in the bioreactor was created by sparging with nitrogen gas at 0.15 vvm during the whole fermentation process.

In fed-batch fermentation, continuous feeding was carried out for the maintenance of the glycerol concentration of 20 g/L during the fermentation period by manually adjusting the feed rate.

### Pure Culture

The four single strains with different morphologies were isolated from the consortium LS30 under microaerobic and anaerobic conditions. Then the pure culture was first purified on solid medium and then cultivated in seed medium A at 37°C. The batch experiments were carried out with seed medium under anaerobic conditions and bioreactor under microaerobic conditions, respectively. The operation condition of pure cultures was same with the consortium.

### Analysis of Microbial Community Structure

The total genomic DNA was extracted from the microbial consortium using EZNA™ Soil DNA Kit (Omega Bio-Tek., Inc., USA) following the manufacturer's instructions and was quantified using a Qubit 3.0 DNA Assay Kit (Life, USA). For each DNA product of microbial consortium, the 16S rRNA V4-V5 region of the bacteria was amplified using the universal primers 515F: 5′-GTGCCAGCMGCCGCGGTAA-3′and 909R: 5′-CCCCGYCAATTCMTTTRAGT-3′. Sequencing was performed on Illumina Miseq sequencing platforms by Sangon Biotech (Shanghai, China). The alignment and analysis of obtained sequences were carried out in the SILVA 16S rRNA gene database.

### Nucleotide Sequence Accession Numbers

The sequences data about microbial consortia in this work have been submitted to the NCBI Sequence Read Archive (SRA) database under accession number SRP158349, SRP158348, SRP158344.

### Analytical Procedures

Microbial growth was monitored by measuring the optical density at 650 nm. During the fermentation, simple measurement of glycerol concentration was performed in terms of Wang et al. (2001). The quantitative analysis of glycerol, 1,3-PDO, LA, acetate, butyrate, and ethanol was achieved by HPLC according to the method of Zhou et al. (2018).

## Results

### Selection of Microbial Consortia

**Figure 1:**
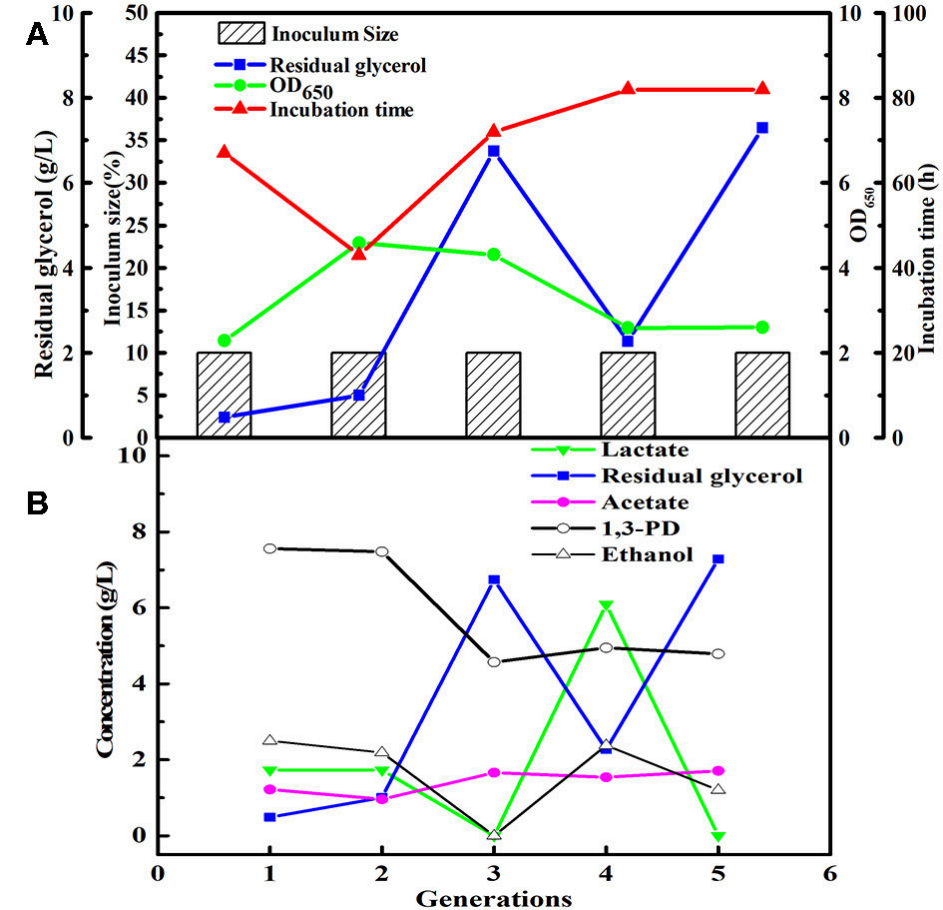
The first five generations of the microbial consortium screened. **(A)** The microbial growth and the conditions for transfer culture. **(B)** The metabolite and residual substrate concentrations vs. transfer generations (6th−30th) using GWCO and seawater.

**Figure 2:**
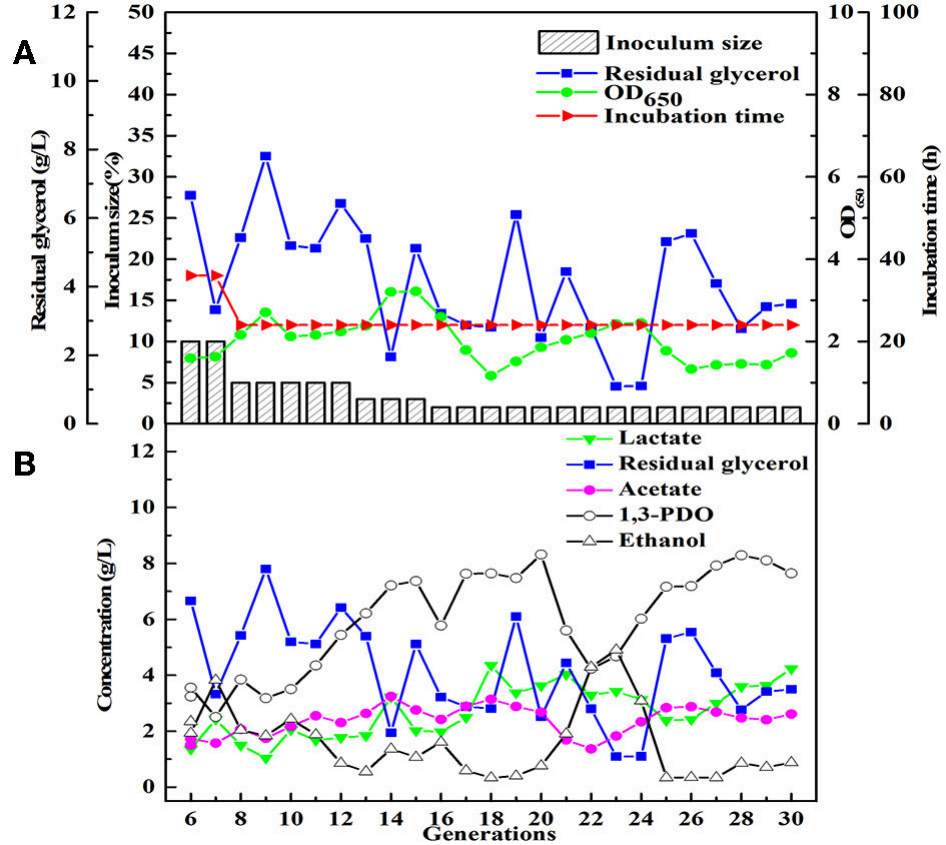
The selection process of the microbial consortium LS30. **(A)** The condition for microbial growth and transfer culture (6–30th). **(B)** He metabolite and residual substrate concentrations vs. transfer generations (6–30th) using GWCO and tap water.

After obtained the activated sludge, an inoculum (5 g) was injected into the seed medium C. When there was a small amount of glycerol surplus in previous culture, the culture broths as the new inoculum were fed into the next bottle contained fresh seed medium. In the initial five transfers, with the increase of transfer times, there was an increase in glycerol surplus, even prolonging the incubation time. Furthermore, the OD value representing the microbial growth decreased (Figures 1A,B). To address this negative situation, four types of seed media (A, B, C, and D) were used for subsequent transfers. The fifth generation of seed broths was inoculated into new seed media A, B, C, and D, respectively. Then, the respective culture broths was transferred to the corresponding medium for 30 times for every microbial consortium. LS30, HG30, SD30, and SG30 represented microbial consortium selected from media A, B, C, and D, respectively. The microbial consortium LS30 mainly produced 1,3-PDO and LA. The by-products included 2.61 g/L acetate and 0.88 g/L ethanol in seed culture (Figures 2A,B). At the initial stage (6th−15th), the microbial consortium LS30 could produce 7.37 g/L 1,3-PDO and 2.02 g/L LA. Accordingly, the OD values also showed an increasing trend, which increased from 1.60 to 3.22 as shown in Figure 2A. The results indicated that the 1,3-PDO producers were enriched during this process. In the next fluctuating stage (16th−24th), the production of 1,3-PDO, LA, and by-products, such as acetate and ethanol were unstable. In this period, microbial abundance and metabolism may be subject to distribution and regulation. In particular, the production of 1,3-PDO decreased rapidly and then increased. On the contrary, the concentration of ethanol showed a reverse trend. Finally, the fermentation performance of the microbial consortium achieved a relatively stable stage (25th−30th) with most parameters such as OD values, 1,3-PDO, lactate, acetate, and ethanol concentrations tend to stable (no more than the concentration difference of 1.5 g/L in the 6 generations). The residual glycerol concentration reached a stable level from the 28th generation.

**Figure 3:**
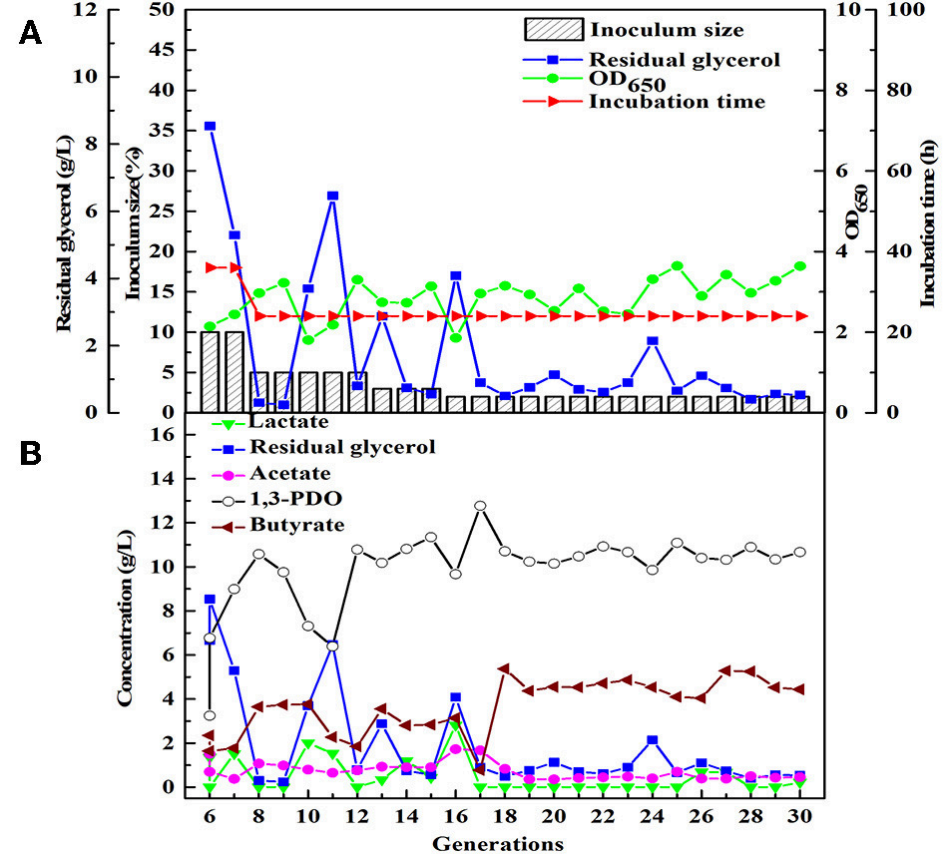
The selection process of the microbial consortium HG30. **(A)** The microbial growth and the conditions for transfer culture (6–30th). **(B)** He metabolite and residual substrate concentrations vs. transfer generations (6–30th) using GHVO and tap water.

Similarly, after three phases of domestication, the microbial consortium domesticated by seed medium B was named HG30. The main product of HG30 is 1,3-PDO (10.67 g/L) accompanied by the production of butyrate and acetate (Figures 3A,B). However, the microbial consortium SD30 and SG30 domesticated by seed medium C, D, respectively, showed relatively weak fermentation performances (see Supplementary Figures 1A,B, 2A,B). As is shown in Table 2, the concentration of 1,3-PDO and LA were 6.15 and 5.60 g/L in the fermentation of microbial consortium SD30 at the initial glycerol concentration of 20 g/L. And the microbial consortium SG30 produced only a small amount of 1,3-PDO (2.73 g/L), ethanol (2.50 g/L), and LA (1.25 g/L).

**Table 2:** Compositions of products of batch flask fermentation in selected microbial consortia culture.

**Inoculum**	**1,3-PDO (g/L)**	**Y_**1, 3-PDO**_ (g _**1, 3-PDO**_/g _**Glycerol**_)**	**Lactate (g/L)**	**Acetate (g/L)**	**Butyrate (g/L)**	**Ethanol (g/L)**
LS30	7.64	0.45	4.23	2.61	–	0.88
HG30	10.67	0.54	0.22	0.45	4.43	–
SD30	6.15	0.36	5.60	1.94	–	1.92
SG30	2.73	0.35	1.25	0.78	–	2.50

*The initial glycerol concentration was 20 g/L*.

### Composition and Diversity of Microbial Consortia

**Figure 4:**
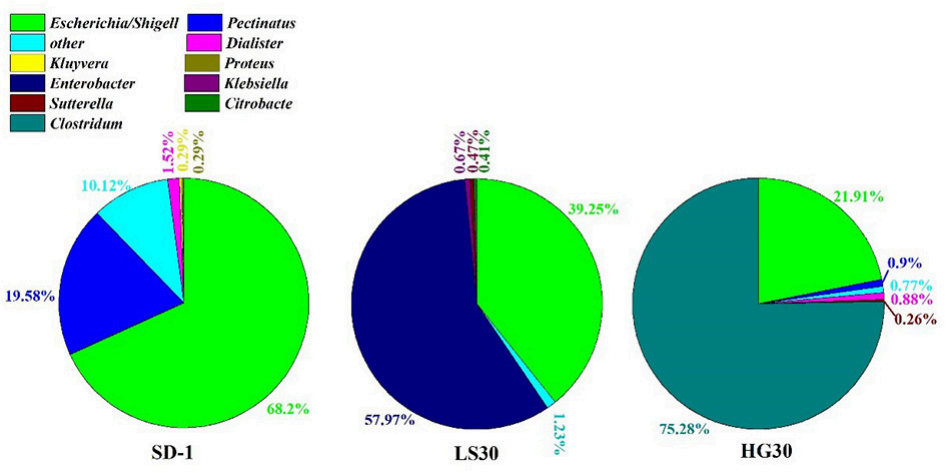
Frequency of genus in the microbial consortium SD-1, LS30, and HG30.

The structural information of three microbial consortia were presented by analysis of high-throughput community sequencing for exploring the change of microbial consortia compositions during the selection processes. The microbial consortium SD-1 from the enrichment of activated sludge samples through the first seed medium was mainly composed of bacteria from *Escherichia/Shigell* and *Pectinatus* genera accounted for 68.2 and 19.58%, respectively (Figure 4). Other genera including *Dialister* (1.52%), *Kluyvera* (0.29%), *Proteus* (0.29*%), Klebsiella (0.01%)* and so on were detected in the microbial consortium SD-1 as well. Furthermore, a relatively large proportion of bacteria (9.3%) in the microbial consortium was not identified. In the microbial consortium LS30, *Enterobacter* (57.97%) and *Escherichia/Shigell* (39.25%) were prevalent genus (Figure 4). *Klebsiella* and *Citrobacter* genera were detected in the consortium, accounting for 0.67 and 0.41%, respectively. By contrast, community structures of the consortium HG30 exhibited simpler than that in LS30, with 75.28% *Clostridium*, 21.91% *Escherichia/Shigella* and 2.33% other genera. A small portion of the unclassified bacteria in the microbial consortium LS30 and HG30 had independently dropped to 0.73 and 0.78%. The change of community structures in the consortia represents an objective domestication result of serial glycerol culture of the initial consortium.

### Batch and Fed-Batch Fermentation of Microbial Consortium LS30

**Figure 5:**
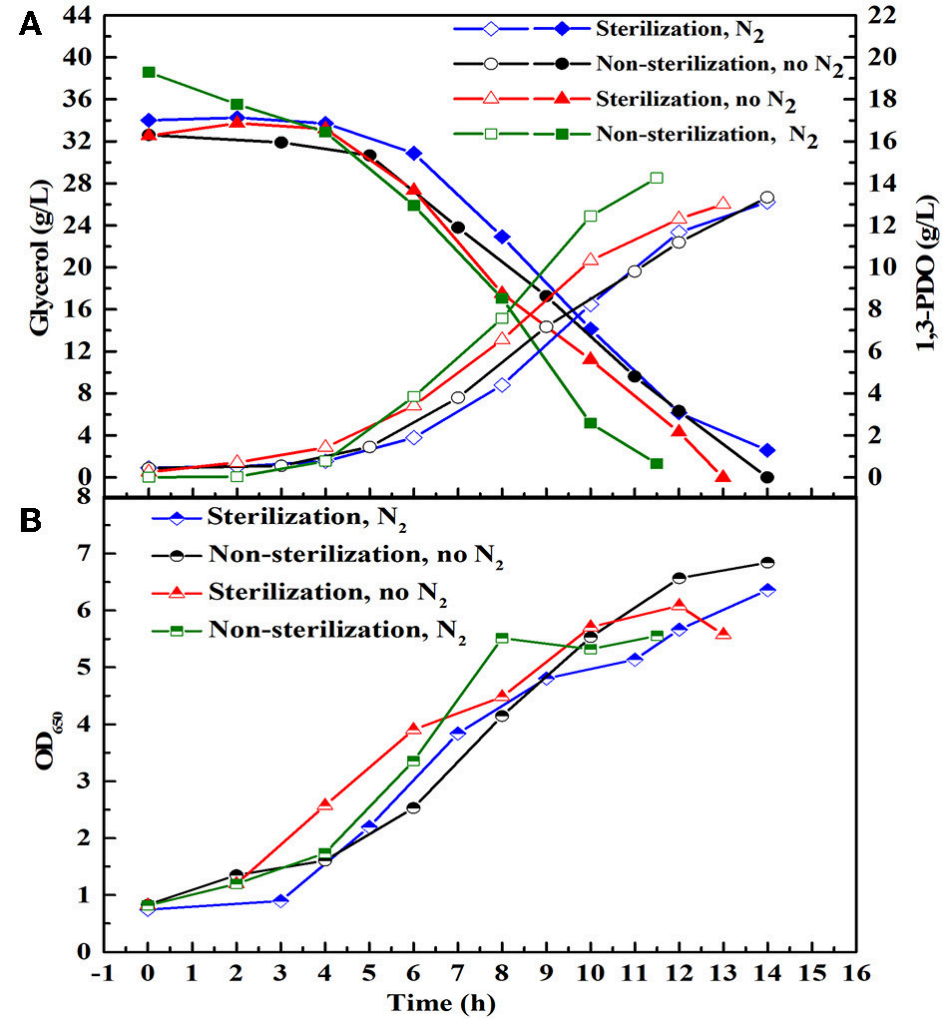
Batch fermentation with 20 g/L initial glycerol under different fermentation conditions. Sterilization and non-sterilization represent sterilized and non-sterilized fermentation medium, respectively. N_2_ means N_2_ (0.15 vvm) was introduced to create anaerobic environment, and No N_2_ means no N_2_ is sparged in the bioreactor and the reactor is in microaerobic condition. **(A)** The glycerol consumption and the production of 1,3-PDO. **(B)** Microbial growth.

There are many reports about the bioconversion of GHVO to valuable chemicals. At the same glycerol concentration (20 g/L), the yield of 1, 3-PDO produced by the consortium HG30 (10.67 g/L) is not superior to that (about 13 g/L) reported by Zhou et al. (2017). The selected consortium LS30 has shown the best tolerance of GWCO and could produce the highest concentration of 1,3-PDO and lactate in the current published reports. Moreover, low price of GWCO makes it more economical for industrial production and its recycle could solve the problem of emission pollution. Therefore, LS30 was used as a consortium to study further. To evaluate the effect of sterile, non-sterile, microaerobic, and anaerobic environment on fermentation behavior of microbial consortium LS30, batch fermentations were conducted with 30 g/L GWCO. As predicted, the main metabolites produced by LS30 were 1,3-PDO and LA along with acetate and ethanol as the major byproducts (Supplementary Table 2). No significant differences in main products yield, glycerol consumption rate (Figure 5A), and microbial growth (Figure 5B) was observed among four different operation conditions. Therefore, the non-sterile and microaerobic working conditions were adopted for further studies because a quite satisfactory fermentation result (13.33 g/L 1,3-PDO, 8.28 g/L LA and carbon recovery of 0.96) was achieved.

**Figure 6:**
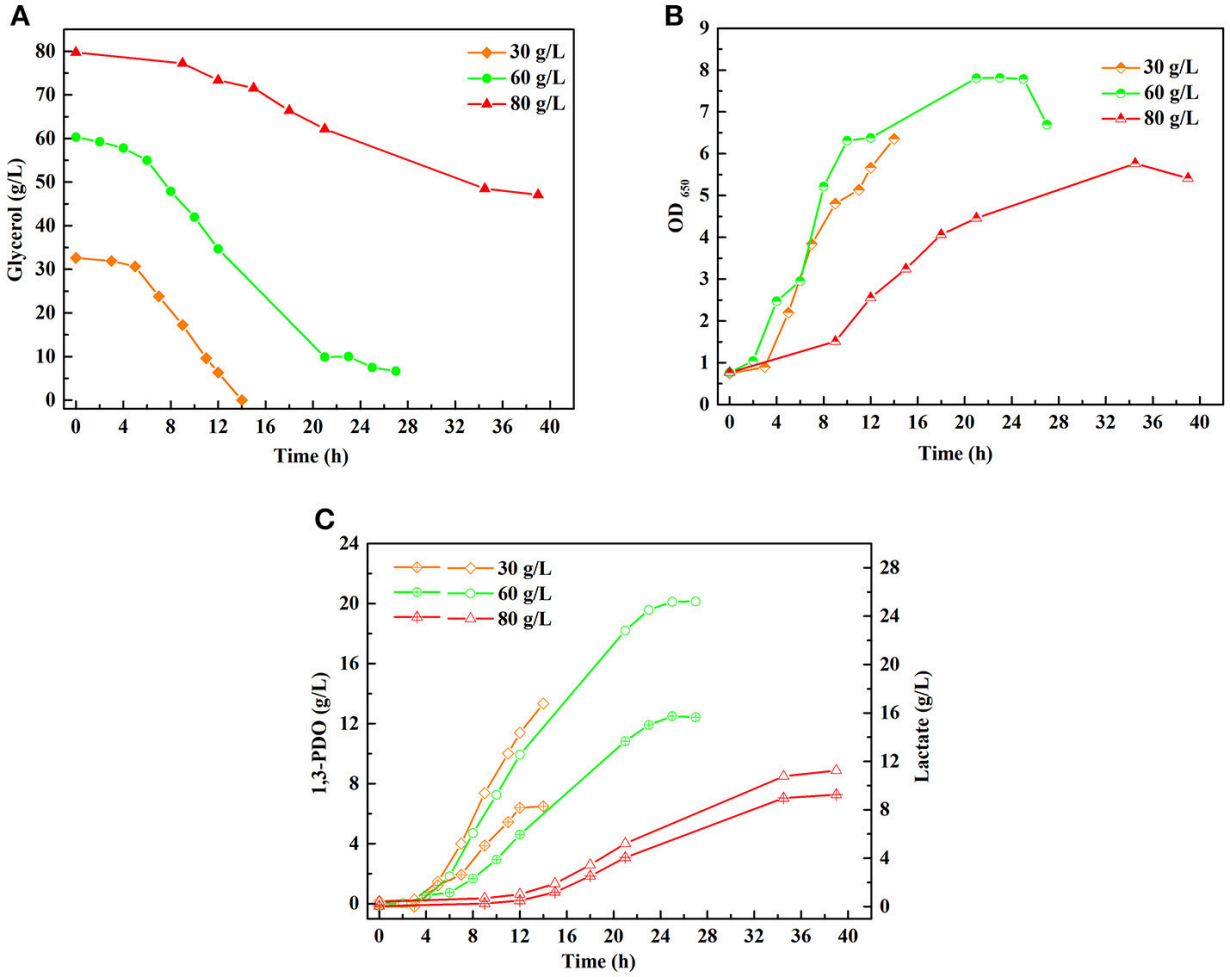
Batch fermentations of microbial consortium LS30 using glycerol from waste cooking-oil-based biodiesel production. **(A)** Glycerol consumption. **(B)** Microbial growth. **(C)** 1,3-PDO and LA production. In **(C)**, the hollow symbol represents the generation of 1, 3-PDO and the line in symbol represents LA production.

**Figure 7:**
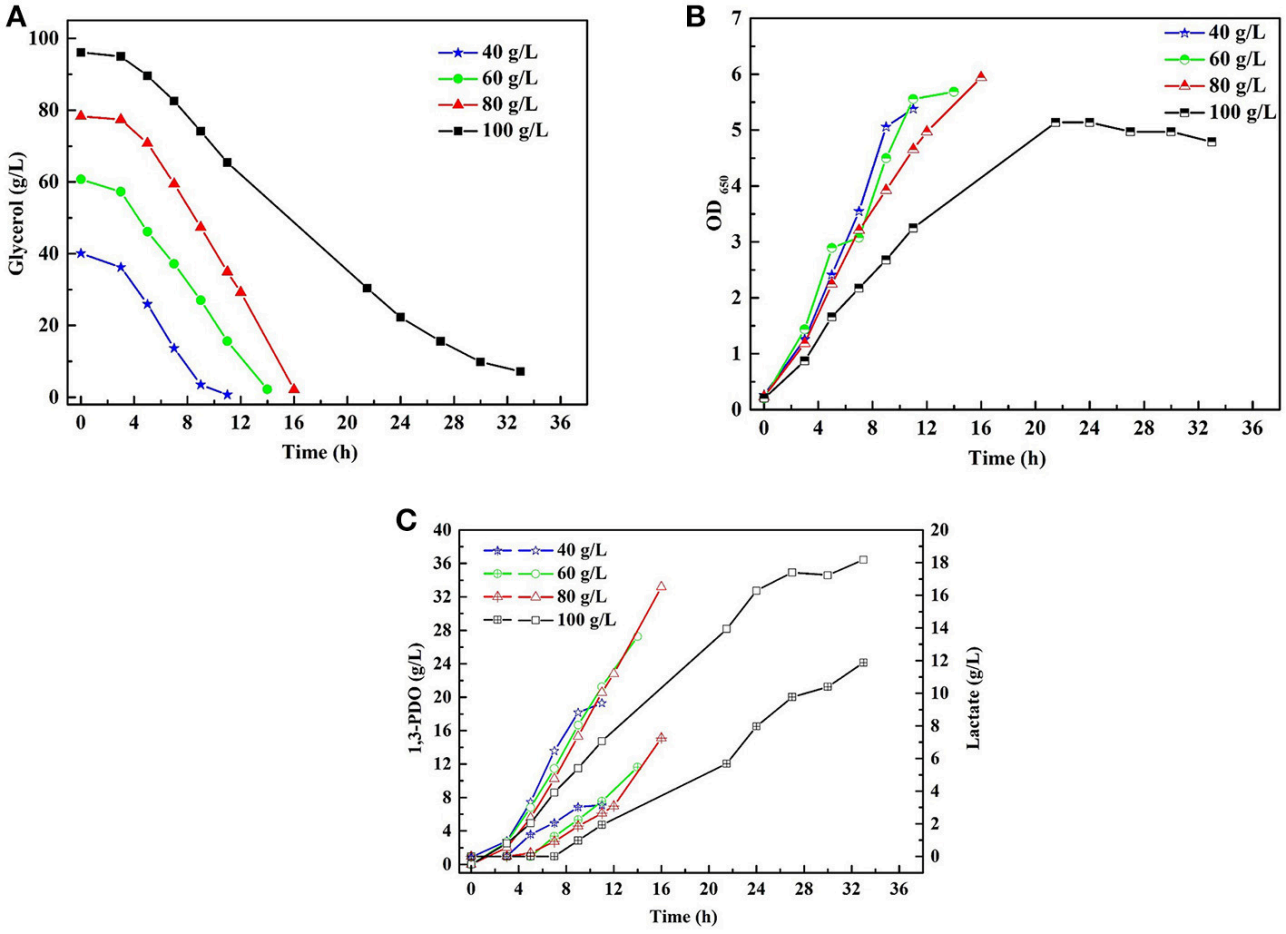
Batch fermentations of microbial consortium LS30 using crude glycerol from hydrolysis of vegetable oils. **(A)** Glycerol consumption. **(B)** Microbial growth. **(C)** 1,3-PDO and lactate production. In **(C)**, the hollow symbol represents the generation of 1, 3-PDO and the line in symbol represents LA production.

A series of batch fermentations under higher initial substrate concentrations were carried out to determine the GWCO tolerance of consortium LS30. When GWCO as the substrate, the highest GWCO consumption for the microbial consortium LS30 was up to 60 g/L under non-sterilize and microaerobic conditions, producing 20.25 g/L 1,3-PDO and 7.70 g/L LA (Figures 6A,C). Above this concentration, the GWCO consumption and microbial growth seemed to be significantly inhibited (Figures 6A,B). In the case of using GHVO as the substrate, microbial consortium LS30 performed a better glycerol tolerance (100 g/L) than using GWCO. As is shown in Figure 7, the consortium LS30 completely consumed 100 g/L glycerol with 33 h, which is much longer than that with 80 g/L (16 h), along with lower highest OD_650_ value. In this process, the consortium LS30 could produce 36.45 g/L 1,3-PDO and 11.87 g/L LA.

**Figure 8:**
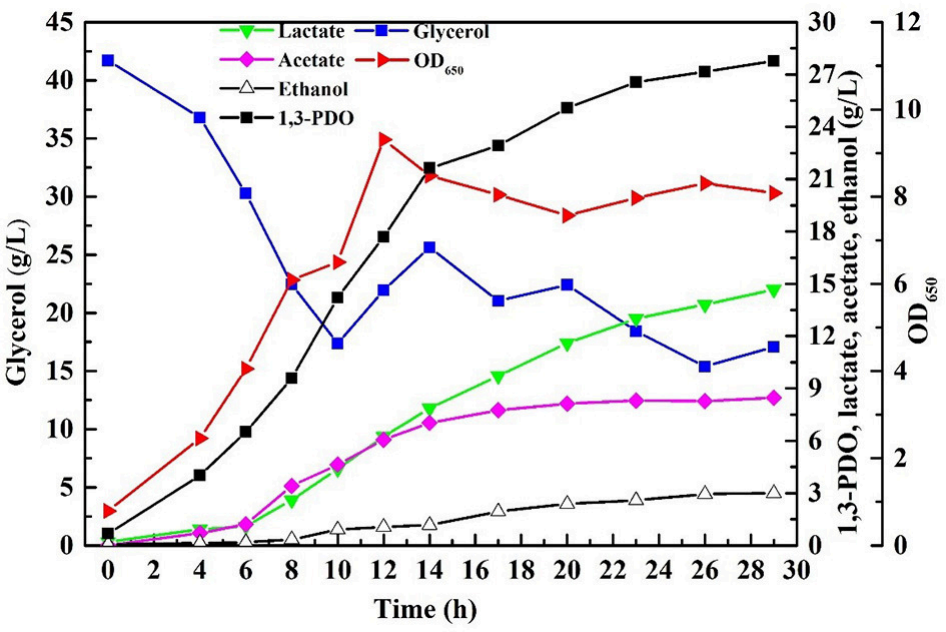
The products and microbial growth in continuous fed-batch fermentation by microbial consortium LS30 using GWCO. The residual glycerol concentration of 20 g/L was maintained by manual adjustment.

Fed-batch fermentation was conducted necessarily with the consortium LS30 for pursuing a higher final products concentration as well as avoiding inhibition of the initial glycerol concentration and other toxic impurities. In fed-batch fermentation, GWCO was fed by continuous feeding strategy when the residual glycerol concentration decreased from initial 40–20 g/L. Then the residual glycerol concentration of 20 g/L was maintained by manually controlling the glycerol feeding rate until the end of fermentation. 65.10 g/L glycerol was consumed in the continuous fed-bath fermentation by the microbial consortium LS30. The fermentation results were shown in Figure 8. The final 1,3-PDO and LA titer were 27.77 and 14.68 g/L, respectively, with the production of the acetate and ethanol. Acetate was mainly produced in the early stage of fermentation (before 14 h), but almost not in the late stage. In addition, contrary to the production of large amounts of acetate (8.47 g/L), the concentration of ethanol hardly increased compared with batch fermentation. Compared with batch fermentation, the 1,3-PDO concentration increased by 24.8% and the increase of LA was remarkable (90.6%).

### Comparison of Glycerol Utilization by Single Strain and Microbial Consortia

**Figure 9:**
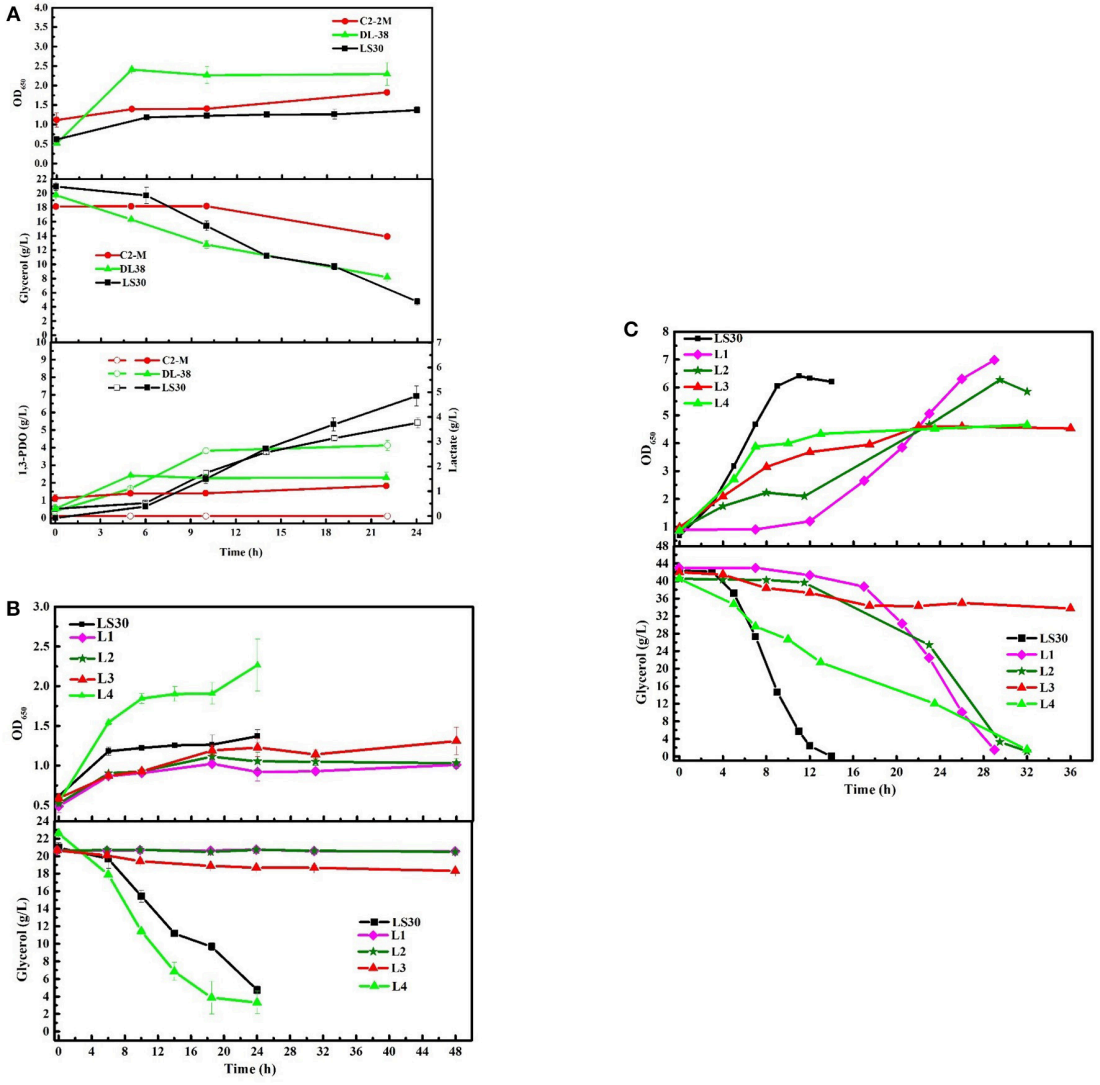
Microbial growth and main products production of microbial consortium LS30, C2-2M, DL38 and four single colonies using raw glycerol from waste cooking based biodiesel production. **(A)** Seed cultures of microbial consortium LS30, C2-2M and DL38 with initial glycerol concentration of 20 g/L. **(B)** Seed cultures of four single colonies and microbial consortium LS30 with initial glycerol concentration of 20 g/L. **(C)** Batch fermentations by four single colonies L1–L4 with initial glycerol concentration of 40 g/L. In the figure, the internal solid symbol represents 1,3-PDO production and the hollow symbol represents the generation of LA. Values are means of two independent fermentation.

To assess the unique strengths of the consortium LS30, the fermentation performance of four single strains isolated from the consortium LS30 (L1-L4) was evaluated. In seed cultures, none of the four single strains was better than the consortium LS30 in production and glycerol tolerance capacity (Figures 9B,C). There were a minimum glycerol consumption with single strain L1, L2 and the limited glycerol consumption capacity of single bacteria L3. As is shown in Figure 9B and Supplementary Table 3, although rapid growth and glycerol utilization were found in strain L4, the production level of each product was quite lower than that of community LS30 (6.93 g/L 1,3-PDO and 3.76 g/L LA). In batch fermentations, when the glycerol concentration was 40 g/L, the growth of each of the four strains was severely inhibited and a very long lag period of glycerol consumption (14 h) appeared in the L1 and L2 cultures. The fermentation time of single strain L1, L2, and L4 was more than twice of that of the consortium LS30, and strain L3 still showed the limited glycerol metabolism (Figure 9C). The products disparity occurred between the consortium LS30 and four single strains. As shown in Supplementary Table 4, the highest concentration of 1,3-PDO (20.96 g/L) and LA (7.78 g/L) were obtained by the consortium LS30, following by isolated strain L2 (10.51 g/L 1,3-PDO and 8.03 g/L LA), strain L4 (7.63 g/L 1,3-PDO and 6.21 g/L LA) and strain L1 (4.73 g/L 1,3-PDO and 4.17 g/L LA). In addition, the primary product of strain L1 was ethanol (11.78 g/L).

The consumption of GWCO was evaluated with two previously reported microbial communities C2-2M (Zhou et al., 2017) and DL38 (Jiang et al., 2017a) considering their high glycerol tolerance and 1,3-PDO production capacity. In seed cultures, the consortium DL38 showed the highest growth rate, whereas the OD_650_ value of consortium LS30 was the lowest (Figure 9A). In terms of products generation, the consortium DL38 mainly produced LA (2.85 g/L), followed by 1,3-PDO (2.29 g/L), whereas the yield of both were far lower than that of consortium LS30. What's worse, consortium C2-2M could hardly consume this low-quality raw glycerol.

## Discussion

### Selection and Community Structure Analysis of Microbial Consortia

Activated sludge contains complex microbial community which could well-adapt to extreme environments and produce industrial products from waste organic matters (Poleto et al., 2016). Based on this feature, four microbial consortia were selected from the activated sludge after 30 serial transfers with different seed media. After the three stages (enrichment, fluctuation, and stabilization) as was described in the Zhou et al. (2017), all four microbial consortia had a potential of the degradation of raw glycerol.

Microbial diversity analysis based on 16S RNA sequence was executed in the first domesticated generation of SD-1, the thirtieth domestication LS30 and HG30. The higher Shannon index indicates the high microbial diversity in the microbial consortium (Ratti et al., 2015). Apparently, in the analysis of the composition of three consortia, the microbial consortium SD-1(Shannon index of 1.18) showed greater microbial diversity, which had been domesticated once with seed medium (Supplementary Figure 3). In contrast, the low community diversity (Shannon index 0.91 and 0.91, respectively) was exhibited by the microbial consortium LS30 (*Enterobacter* of 57.97% and *Escherichia/Shigell* of 39.25%) and HG30 (*Clostridium* of 75.28%, *Escherichia/Shigella* of 21.91%) after 30 serial transfers. The selection process indicated that the proportion of *Enterobacter* and *Clostridium* in the consortium SD-1 were too small to be detected, but after enrichment and adaptation, the consortia LS30 and HG30, mainly composed of *Enterobacter* and *Clostridium*, respectively, were obtained. *Enterobacter* and *Clostridium* are classical genus to convert glycerol to valuable products (Chanthoom et al., 2016), *Enterobacter* mainly produces ethanol, hydrogen, and 1,3-PDO (Barbirato et al., 1995; Pachapur et al., 2015; Lee et al., 2017), *Clostridium* is a superior 1,3-PDO producer (Wilkens et al., 2012; Zhou et al., 2017, 2018). These results showed that the screening process is effective and necessary for enriching the required microorganisms. In selection process, microorganisms which could adapt seed cultures were enriched rapidly, but other microorganisms that could not endure the seed cultures or grew slowly were removed. However, the differences in composition between the consortium LS30 and HG30 is mainly due to the fact that microorganisms response differently on impurities in two kinds of glycerol. It is suggested that *Enterobacter* has better resistance to GWCO than *Clostridium*, so it can be preserved in consortium LS30 by serial transfers. According to Pachapur et al. (2016) and Rodrigues et al. (2018), *Enterobacter* could convert GWCO to hydrogen with a small amount of 1,3-PDO. On the contrary, *Clostridium* exhibited weak tolerance to low-quality glycerol with complex impurities (Moon et al., 2010). As a result, this type of microorganism did not appear in the community LS30, but in consortium HG30 with considerable proportion. It was also indicated *Clostridium* exhibited competitive degeneration capacity for GHVO could be rapidly and abundantly enriched, while *Enterobacter* was eliminated because of its relatively slow growth. Notably, *Escherichia* was present in all three consortia. According to Dharmadi et al. (2006) and Murarka et al. (2008), *Escherichia* was found to anaerobically consume glycerol in the absence of electron acceptors, which renews the conventional knowledge that *Escherichia* has no ability of glycerol consumption when there are no external electron acceptors. In their studies, *Escherichia* could grow in seed medium contained little nutrients (2 g/L tryptone or 5 g/L corn steep liquor) and the supplementation of CO_2_ could accelerate the consumption of glycerol. Only 0.1 g/L glycerol was used for microbial growth, but there were no cell growth when the glycerol was not present in medium. Therefore, the possible explanations of *Escherichia* presented in three selected consortia could be divided into three aspects. Firstly, *Escherichia* can be well adapted to many harsh environments (high salinity, a small amount of nutrients or many impurities) with less glycerol consumption. Secondly, CO_2_ produced by *Enterobacter* and *Clostridium* from glycerol metabolism exists in culture bottle so as to enhance growth of *Escherichia*. Lastly, microbial interactions occurred in consortia such as electron acceptors of *Escherichia* played by *Clostridium* or *Enterobacter*.

As for the consortium HG30, its composition is different from that of the consortium C2-2M (94.64% *Clostridiaceae* and 4.47% *Peptostreptococcaceae*) reported by Zhou et al. (2017). The differences may depend on whether L-cysteine · HCl is added to the seed medium. L-cysteine · HCl, a deoxidant in the seed medium, may create a perfect anaerobic environment for rapid enrichment of strictly anaerobic *Clostridiaceae*. Meanwhile, other microorganisms with small proportion were eliminated during the serial transfer process. Furthermore, it is an understandable result that both community SG30 and SD30 cannot effectively use the two kinds of glycerol to produce products. After all, the extremely halophilic bacterium *Salinibacter ruber* can convert 40% of the amount of glycerol added to the medium to CO_2_ and 12% substrate consumption was incorporated by cells (Oren and Mana, 2003). Therefore, the production of CO_2_ may be the main cause of carbon loss. Another reason is that activated sludge samples may contain little halophilic bacteria. A few reports are about glycerol metabolism by halophilic bacteria, and the growth of experimental microbial consortia were indeed inhibited by the salinity environment with enormous osmotic pressure.

### Unique Fermentation Performance of Microbial Consortium LS30

The batch fermentation under different conditions indicated that the microbial consortium LS30 could accomplish the glycerol fermentation under microaerobic and non-sterile conditions. *Enterobacter* and *Escherichia*, as dominant bacteria in the community LS30, are facultative anaerobic bacteria (Kumar et al., 2014). A possible fermentation pattern was predicted, where at the beginning of fermentation, it was conductive to the microbial growth under aerobic conditions and some bacteria could consumed little glycerol with quick oxygen depletion. Then 1, 3-PDO was produced under anaerobic conditions. Microbial consortium can perform complex task with their robustness to environment fluctuations (Brenner et al., 2008). Therefore, microbial consortium LS30 can maintain steady fermentation performance under four fermentation conditions.

A series of batch fermentations were performed with GWCO (30–60 g/L) and GHVO (40–100 g/L) (Figures 6A, 7A). The results indicated that the microbial consortium LS30 was unable to tolerant high concentration of GWCO but can consume completely a higher concentration of GHVO (100 g/L). It showed that specific impurities in the low-quality glycerol, but not glycerol concentration, had serious inhibitory effects on the growth of microorganisms. The composition of GWCO was shown in Table 1 and Table 2. The raw glycerol does contain many impurities such as soaps (12.10%), salts (9.72%), aflatoxin B1 (2.5–5 ppb), methanol (1.2%), acetate (3.9%), 1,3-PDO (3.8%) and heavy metal. Different amounts of methanol and soap in GWCO have been reported by Rodrigues et al. (2018) and the soluble soap had been removed from crude glycerol by pretreatment of hydrochloric acid (Rodrigues et al., 2018). The content and variety of impurities in GWCO depended on the character of WCO and the process of biodiesel production. The presence of stable content of acetate and 1,3-PDO in GWCO probably came from WCO, where bacteria used sugar or waste glycerol to produce these two chemicals. The existence of soap especially the presence of double bond in it may be the most potent inhibitors for the growth behavior of 1,3-PDO producers (Chatzifragkou et al., 2010; Anand and Saxena, 2012). A large number of uncertain impurities did inhibit the growth and fermentation properties of the microbial consortium LS30. In batch fermentation, the consortium LS30 could produce 1,3-PDO and LA with a maximum concentration of 20.25 and 7.70 g/L, respectively.

It was indeed the presence of a mass of impurities rather than glycerol concentration that hampered microbial growth in batch fermentation. According to the results of Szymanowska-Powałowska and Kubiak (2015), toxic compounds were added to the medium before inoculation and at microbial exponential growth, and the results indicated *C. butyricum* DSP1 has a stronger adaptability for the latter condition. Therefore, fed-batch fermentation was performed. In a fed-batch fermentation, continuous feeding strategy was adopted to maintain the glycerol concentration of 20 g/L when initial glycerol was 40 g/L. As expected, a higher concentration of 1,3-PDO (27.77 g/L) and LA (14.68 g/L) were obtained. The 1,3-PDO and LA concentration increased by 24.8 and 90.6%, respectively, compared with batch fermentation. Of course, the 1,3-PDO and LA concentration cannot compete with other reports, such as 70 g/L 1,3-PDO in Dietz and Zeng (2014), 82.66 g/L1,3-PDO in Zhou et al. (2017), because GWCO is used as the substrate and the definite impurities in it inhibited the growth of bacteria indeed.

### Advanced Impurities Adaptability of Microbial Consortium LS30

In order to verify that the selected microbial consortium has the advanced adaptability to GWCO, batch fermentations were carried out using isolated strains from the consortium LS30. The seed cultures with four pure strains demonstrated that only the strain L4 was as capable of consuming glycerol as the consortium LS30. Unfortunately, the single colony L4 produced a fewer amounts of 1,3-PDO (3.42) and LA (2.08 g/L) than original community (Supplementary Table 3). When the initial glycerol concentration was up to 40 g/L, a very long growth delay existed in pure cultures, which caused its fermentation time to be twice as long as that of the original consortium. The different fermentation performance of strain L1 and L2 under batch flask and fermentations is closely related to the composition of culture medium and culture conditions. In our experiments, an irritating odor was produced from the sterilized medium. The unknown toxic gas in sealed serum bottle may have a serious inhibitory effect on the two strains. And there was only 1 g/L of yeast extract as nutriment for the strains growth. However, in batch fermentations, the two strains were breed by non-sterilized fermentation media in microaerobic condition. In this case, there is no inhibiting gas and 2 g/L of yeast extract as nutriment for the strains growth. Therefore, after a long period of adaptation, the two strains began to consume glycerol rapidly. All individual bacteria had different product distribution with the consortium. Microbial consortium exhibited a higher efficiency or productivity even glycerol tolerance than single strain isolated from its consortium (Jiang et al., 2017a; Zhou et al., 2017). The microbial consortium can balance a lot of works efficiently and perform functions that single strain may pose insurmountable challenges under the particular situation (Brenner et al., 2008). Briefly, the microbial consortium LS30 had the advanced adaptability to GWCO and could effectively transform GWCO into valuable products. These achievements were attributed to the cooperation and communication among the different individuals.

Using GWCO as substrate, batch fermentations were also performed using the microbial consortium DL38 with excellent glycerol tolerance (200 g/L) and consortium C2-2M with high yield of 1,3-PDO (82.66 g/L). The consortium C2-2M, in which, *C. butyricum* was the dominant species, grew slowly and cannot convert glycerol to 1,3-PDO effectively as reported by Zhou et al. (2017). Likewise, the consortium DL38, in which, *Enterobacteriaceae* (95.57%) was the main species, could not produce 1,3-PDO as the main product but a higher concentration of LA (2.85 g/L) was identified, although the normal growth of bacteria and complete consumption of GWCO were observed. These also proved that *Clostridium* possesses a relatively weak adaptability to impurities in glycerol and was eliminated in the consortium LS30 selection process. While, the consortium LS30 can tolerate the GWCO and had higher conversion rates of glycerol than DL38 and C2-2M. These results demonstrated that the consortium LS30 had unique advantages in using GWCO and producing products.

In conclusion, a microbial consortium LS30 consisted of 57.97% *Enterobacter* and 39.25% *Escherichia* was selected from activated sludge by 30 serial transfers. The consortium LS30 could convert GWCO to 1,3-PDO and LA under microaerobic and non-sterile conditions. Despite the presence of impurities in glycerol (aflatoxin B1, soap, salts, metal irons, methanol and acetate), the microbial consortium LS30 showed high ability of coproduction of 1,3-propanediol (1,3-PDO) and LA. The highest 1,3-PDO concentration of 27.77 g/L and 14.68 g/L LA were achieved in a fed-batch fermentation with continuous feeding strategy. The microbial consortium LS30 exhibited unique advantages for conversion of GWCO to value-added 1,3-PDO and LA compared with the other consortia such as DL38, C2-2M as well as isolated single strains. The collaborative effect and information communication among single strain could make the consortium better adapt GWCO and produced products efficiently. This study has built a significant and functional bridge for integration of most economical biodiesel production industry with higher-value products production by the bio-utilization of its raw glycerol.

## Author Contributions

Z-LX contributed with initial ideas about the project. Z-LX and X-LW conceived the design of the study. X-LW performed simulations. X-LW, J-JZ, and Y-QS participated in the interpretation of initial results. X-LW drafted the manuscript. All authors contributed to the preparation of the final version of the manuscript.

### Conflict of Interest Statement

The authors declare that the research was conducted in the absence of any commercial or financial relationships that could be construed as a potential conflict of interest.
