# Supplementary material for: Bioconversion of Raw Glycerol From Waste Cooking-Oil-Based Biodiesel Production to 1,3-Propanediol and Lactate by a Microbial Consortium
Source: Front Bioeng Biotechnol. 2019 Feb 18;7:14. doi: 10.3389/fbioe.2019.00014 (PMC6387908; doi:10.3389/fbioe.2019.00014)
Supplement: Supplementary file 1 [file Table_1.docx]

***Supplementary Material***

**Bioconversion of raw glycerol from waste cooking-oil-based biodiesel production to 1,3-propanediol and lactate by a microbial consortium**

Xiao-Li Wang, Jin-Jie Zhou, Ya-Qin Sun, Zhi-Long Xiu*

***Corresponding author:** Zhi-Long Xiu: zhlxiu@dlut.edu.cn

# Supplementary Figures and Tables

## Supplementary Tables

Table 1 The total amounts of trace metal elements in the raw glycerol from biodiesel production using waste cooking oil

| Type of metal elements | Contents (μg /g) |
| --- | --- |
| K | 2.049Ⅹ10^2^ |
| Ca | 1.898Ⅹ10^2^ |
| Cu | 1.593Ⅹ10^2^ |
| Mg | 6.130Ⅹ10 |
| Fe | 5.580Ⅹ10 |
| Mo | 4.3665Ⅹ10 |
| Zn | 3.419Ⅹ10 |
| Al | 1.908Ⅹ10 |
| Se | 7.6356 |
| Cr | 7.134 |
| As | 4.1478 |
| Ni | 4.000 |
| Pb | 1.268 |
| Sr | 8.791Ⅹ10^-1^ |
| Mn | 6.866Ⅹ10^-1^ |
| Li | 2.000Ⅹ10^-1^ |
| Rb | 2.000Ⅹ10^-1^ |
| Co | 1.000Ⅹ10^-1^ |
| V | 1.000Ⅹ10^-1^ |
| Be | 1.000Ⅹ10^-4^ |

Table 2 Compositions of product in batch fermentation under different conditions

| Fermentation conditions | Initial glycerol (g/L) | 1,3-PDO (g/L) | Lactate (g/L) | Acetate (g/L) | Ethanol (g/L) | Carbon recovery |
| --- | --- | --- | --- | --- | --- | --- |
| Sterilization, N_2_ | 34.02 | 13.11 | 8.70 | 2.96 | 2.94 | 0.93 |
| Non-sterilization, No N_2_ | 32.61 | 13.33 | 8.28 | 3.59 | 2.21 | 0.96 |
| Sterilization, No N_2_ | 32.54 | 13.01 | 8.60 | 3.54 | 2.71 | 0.98 |
| Non-sterilization, N_2_ | 38.57 | 14.26 | 6.51 | 3.60 | 3.11 | 0.85 |

Table 3 The products in batch flask fermentation by four single strains and the microbial consortium LS30

| Inoculum | Time (h) | Initial glycerol (g/L) | 1,3-PDO (g/L) | Lactate (g/L) | Acetate (g/L) | Ethanol (g/L) |
| --- | --- | --- | --- | --- | --- | --- |
| LS30 | 24 | 20.96±0.54 | 6.93±0.57 | 3.76±0.21 | 2.04±0.18 | 0.84±0.05 |
| L3 | 48 | 20.71±0.15 | -- | -- | -- | 1.09±0.13 |
| L4 | 24 | 22.61±0.17 | 3.42±0.14 | 2.08±0.16 | 0.90±0.33 | 5.65±0.54 |

No glycerol consumption and products by single strain L1, L2 fermentation.

“--” represents no corresponding products.

Table 4 The products in batch fermentations by four single colonies L1-L4 and the consortium LS30 with initial glycerol concentration of 40 g/L

| Inoculum | Time (h) | Initial glycerol (g/L) | 1,3-PDO (g/L) | Lactate (g/L) | Acetate (g/L) | Ethanol (g/L) |
| --- | --- | --- | --- | --- | --- | --- |
| LS30 | 14 | 42.35 | 13.22 | 7.87 | 3.54 | 2.62 |
| L1 | 29 | 42.99 | 4.73 | 4.17 | 0.76 | 11.78 |
| L2 | 32 | 40.48 | 10.51 | 8.03 | 1.28 | 7.09 |
| L3 | 36 | 41.96 | -- | -- | 0.74 | 3.02 |
| L4 | 23.5 | 40.52 | 7.63 | 6.21 | 1.03 | 6.63 |

“--” represents no corresponding products.

## Supplementary Figures


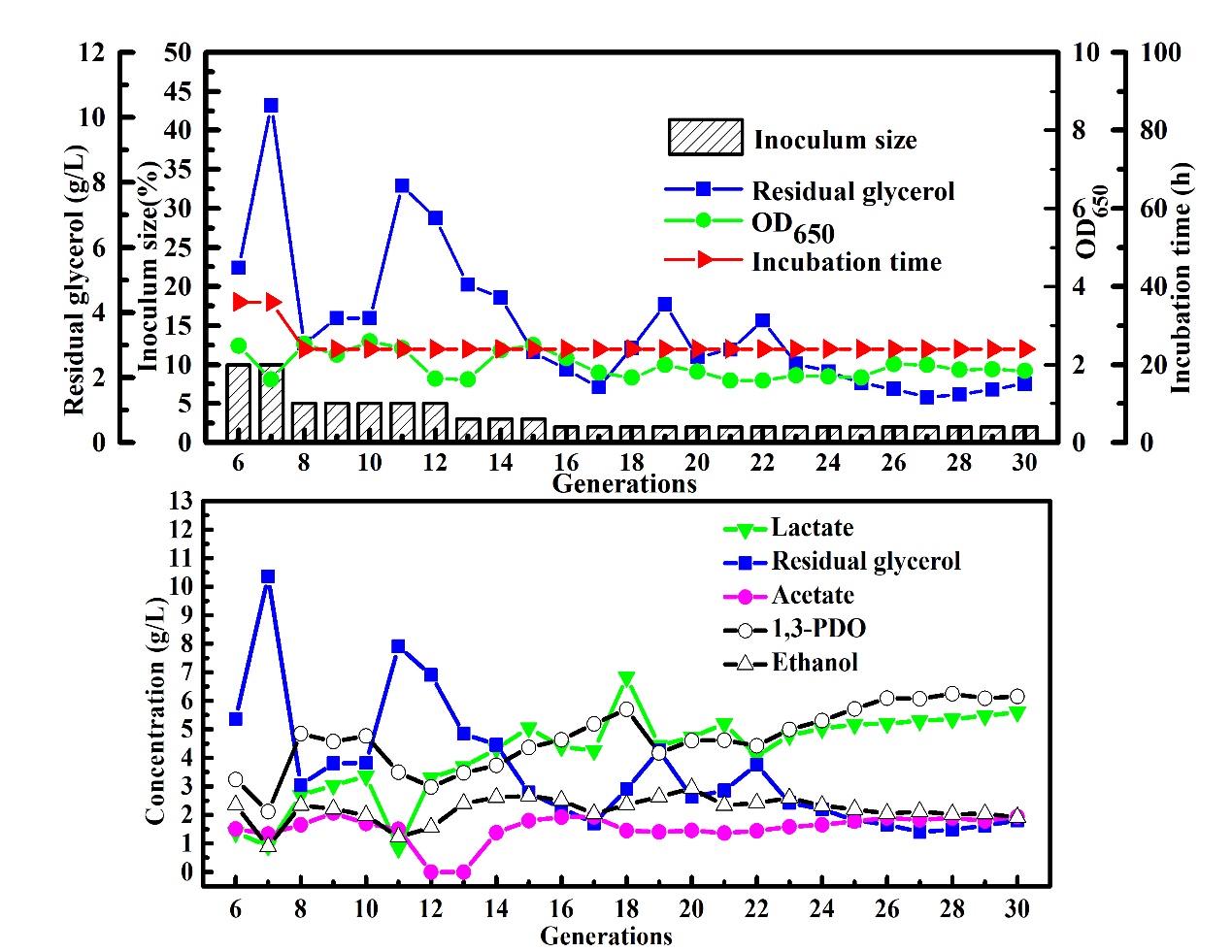


(A)

(B)

**Figure 1** Selection process of the microbial consortium SD30. (A) The microbial growth and the conditions for transfer culture (6-30th). (B) metabolite and residual substrate concentrations vs. transfer generations (6-30th) using GWCO and seawater


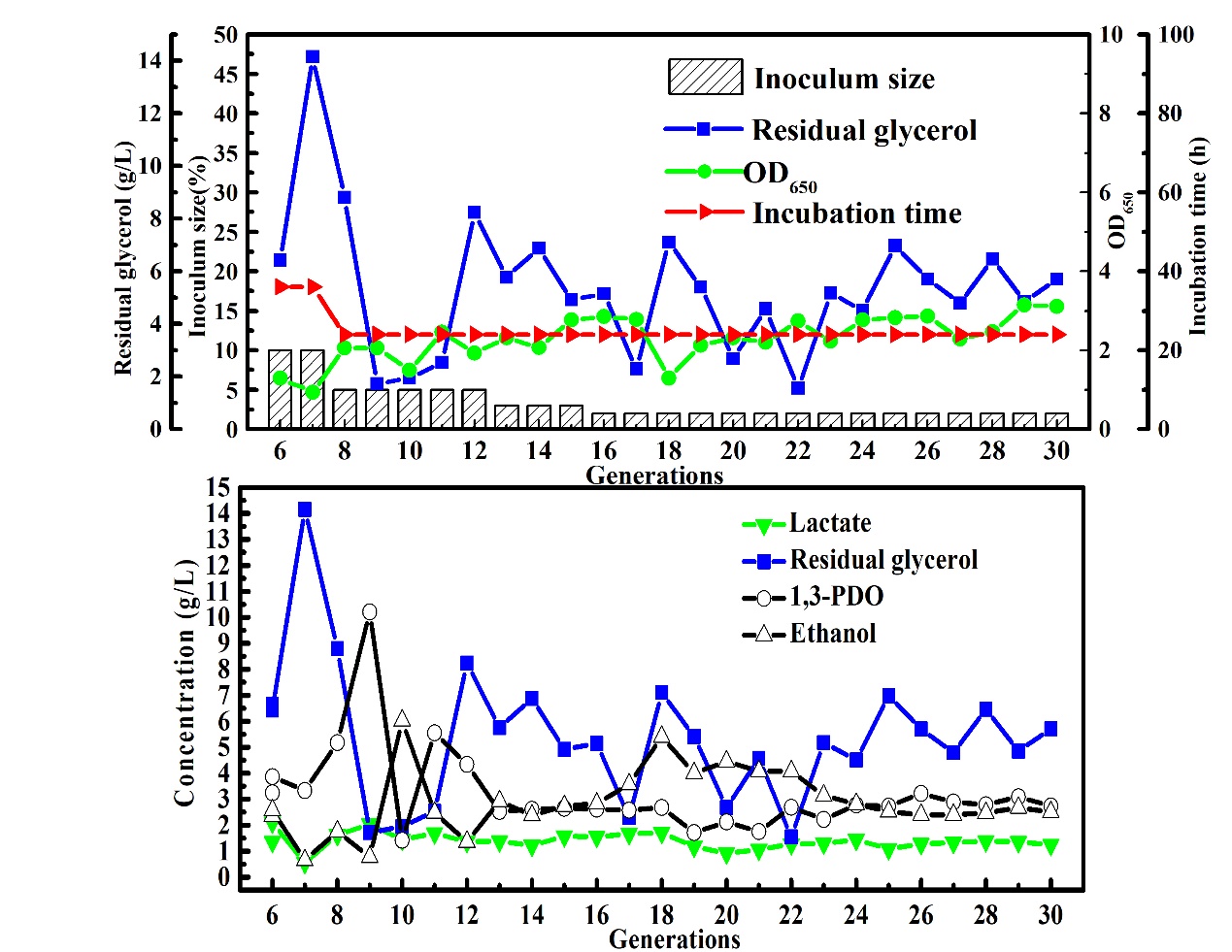


(B)

(A)

**Figure 2** Selection process of the microbial consortium SG30. (A) The microbial growth and the conditions for transfer culture (6-30th). (B) The metabolite and residual substrate concentrations vs. transfer generations (6-30th) using GHVO and seawater (6-30th)


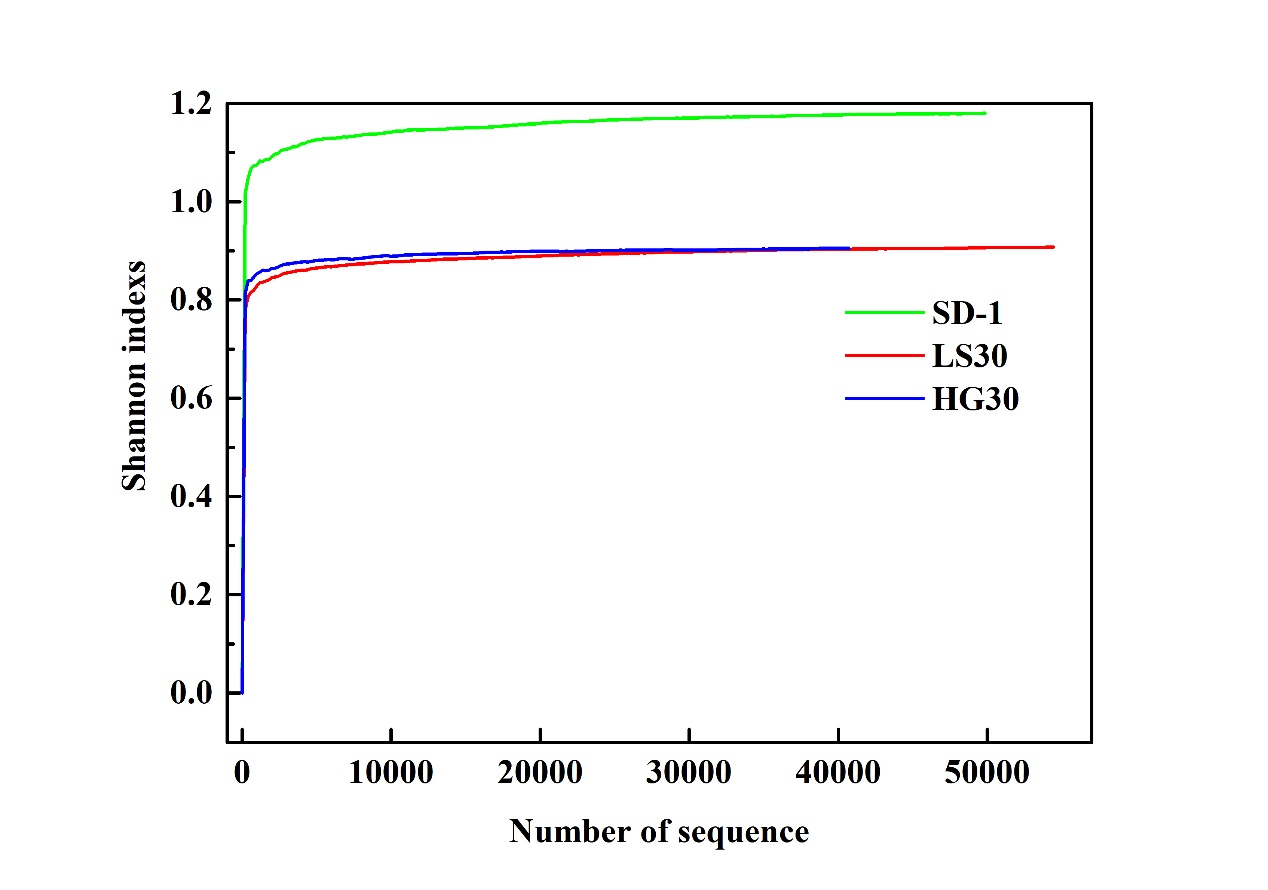


**Figure 3** Shannon rarefaction of microbial consortium SD-1, LS30 and HG30
